# A Hierarchical Neural Network for Point Cloud Segmentation and Geometric Primitive Fitting

**DOI:** 10.3390/e26090717

**Published:** 2024-08-23

**Authors:** Honghui Wan, Feiyu Zhao

**Affiliations:** 1 College of Computer Science, South-Central Minzu University, No. 182 Minzu Avenue, Hongshan District, Wuhan 430074, China; czfeng.w@foxmail.com; 2 Key Laboratory of Cyber-Physical Fusion Intelligent Computing (South-Central Minzu University), State Ethnic Affairs Commission, No. 182 Minzu Avenue, Hongshan District, Wuhan 430074, China

**Keywords:** computer vision, point cloud, segmentation, primitive fitting

## Abstract

Automated generation of geometric models from point cloud data holds significant importance in the field of computer vision and has expansive applications, such as shape modeling and object recognition. However, prevalent methods exhibit accuracy issues. In this study, we introduce a novel hierarchical neural network that utilizes recursive PointConv operations on nested subdivisions of point sets. This network effectively extracts features, segments point clouds, and accurately identifies and computes parameters of regular geometric primitives with notable resilience to noise. On fine-grained primitive detection, our approach outperforms Supervised Primitive Fitting Network (SPFN) by 18.5% and Cascaded Primitive Fitting Network (CPFN) by 11.2%. Additionally, our approach consistently maintains low absolute errors in parameter prediction across varying noise levels in the point cloud data. Our experiments validate the robustness of our proposed method and establish its superiority relative to other methodologies in the extant literature.

## 1. Introduction

Recent advancements in three-dimensional (3D) scanning technology, coupled with increased data storage capacity, have significantly impacted the field of computer vision, offering unparalleled opportunities for the processing and analysis of 3D geometric data. Nevertheless, the scanned data often exists in rudimentary forms, such as point clouds or meshes, which lack direct expressions of structure and semantic information inherent to objects [1]. This poses a notable challenge for precise 3D object manipulation, as it hampers the understanding and reproduction of an object’s structural and semantic integrity. These challenges are acutely felt in advanced editing and model-fitting tasks, where the absence of well-defined geometric primitives and their associated parameters obstructs the achievement of desired outcomes. Consequently, researchers are assiduously converting this raw data into organized, semantically rich representations—a data enhancement process that is crucial for computer vision and serves as a foundational step towards achieving accurate digital twins of physical entities. The importance of this data enhancement process has been emphasized in the fields of Constructive Solid Geometry (CSG) [2] and optical sensor fusion [3], which contribute to 3D reconstruction. CSG is the foundation of Computer-Aided Design (CAD), further highlighting the need for such data enhancement.

In the domains of computer vision and machine learning, the RANSAC [4] algorithm is widely employed, particularly for the fitting of geometric primitives, including lines, circles, and planes. RANSAC excels at selecting the minimum subset of points from a dataset required to define a model’s parameters. Distinctly, RANSAC operates without initial knowledge of the outlier ratio within the data and demonstrates considerable resilience to outliers, often successfully estimating a reasonable model amidst substantial noise and outliers. Nevertheless, RANSAC-based fitting techniques can demand significant computational resources, particularly with vast datasets or when the ratio of inliers to outliers is low. Numerous iterations may be essential to ascertain the optimal model parameters. Furthermore, the algorithm is stochastic, resulting in varying outcomes across different executions due to the random selection of points in each iteration. It is also sensitive to the specification of parameters like the inlier threshold and iteration count, where improper configurations can yield suboptimal fitting results. In extreme cases where outliers overshadow inliers, RANSAC might converge to a local optimum without ensuring a global optimum, rendering it unsuitable for processing extensive point cloud data.

In contrast to conventional primitive-fitting methods, the Supervised Primitive Fitting Network (SPFN) [5] framework introduced by Li et al. [5] leverages the PointNet++ [6] architecture as an encoder for processing input point clouds. This network can autonomously determine the relationships between points and primitives, discerning their types and deducing the parameters that minimize fitting error, which markedly diminishes reliance on manual parameter adjustment and initial processing stages. Thanks to the deep network’s robust performance, SPFN excels at feature extraction from intricate datasets and managing complex environments that pose a challenge to traditional algorithms. Nevertheless, SPFN’s efficacy is limited by PointNet++‘s inherent processing capacity, leading to difficulties in fitting small-sized primitives and handling high-resolution point clouds. To address these constraints, researchers such as Lê et al. [7] have improved upon SPFN by introducing the Cascaded Primitive Fitting Networks (CPFNs) [7] framework, enhancing the matching of diverse primitive sizes with high-resolution point clouds. Despite these advancements, fundamental issues persist, as the networks continue to utilize PointNet++ and exhibit limitations in point feature extraction and primitive fitting.

**Figure 1:**
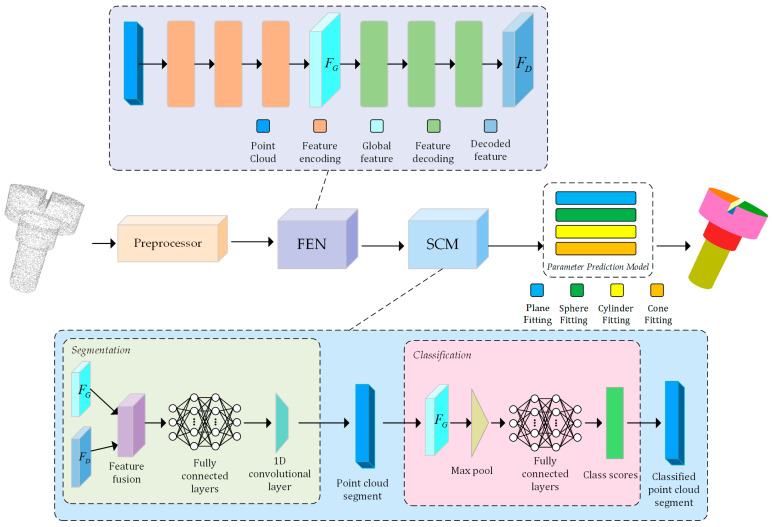
Architecture diagram. It is mainly composed of four modules, namely the preprocessing module, the feature extraction module, the segmentation and classification module, and the parameter prediction module.

In this study, we propose a novel architecture, the details of which are depicted in Figure 1.

The initial step in our research involves de-noising and re-sampling the input point cloud data through our preprocessing module. This preprocessing not only improves the quality of the data but also lays the foundation for subsequent feature extraction. Following the preprocessing, the feature extraction network mines rich geometric features from these refined point clouds, thus providing support for in-depth geometric shape analysis and understanding. The effectiveness of this network is demonstrated in its ability to capture the essential geometric information in the point cloud data, which is an indispensable step in the entire primitive-fitting process. After feature extraction, the segmentation and classification module further process these features, effectively partitioning the entire point cloud into multiple subsections and accurately categorizing each section into the corresponding geometric primitive types. This module employs advanced machine-learning techniques capable of discerning and categorizing various geometric structures within the point cloud, thereby ensuring accuracy and efficiency in subsequent processing steps. Following meticulous segmentation and accurate classification, the parameter prediction module is responsible for precise parameter estimation of each segmented primitive. This module utilizes a weighted least squares method to infer the geometric parameters of the primitives, thus achieving precise fitting of the geometric primitives to the point cloud data.

The primary contributions of our research can be summarized as follows:

- This study introduces an innovative feature extraction network that is specifically designed to address the issue of imprecision that arises during the process of geometric primitive recognition.
- We propose a parameter prediction module, the design of which is intended to predict the parameters of geometric primitives more accurately. This is crucial for enhancing the precision of shape representation and for the understanding of complex 3D structures, marking an important step toward fine-grained 3D modeling.
- Extensive experimental validation demonstrates that our method shows significant advantages in terms of accuracy. These experimental results fully substantiate the effectiveness and practicality of our model in the processing of 3D geometric data, providing reliable technical support for further research and practical applications in the field of computer vision.

## 2. Related Work

Three-dimensional point cloud data inherently lack a direct representation of object structure and semantic information, a trait that poses a significant obstacle to precise three-dimensional object manipulation within the field of computer vision [1]. This limitation constrains the ability to discern and replicate the structural and semantic characteristics of objects from the data. The constraint is particularly pronounced during complex tasks such as advanced editing and model fitting. Due to the absence of clearly defined geometric primitives and their associated parameters, achieving the anticipated results becomes increasingly challenging.

In the field of unstructured data extraction, such as recovering or extracting CSG representations of constructed entities from point clouds, 3D point cloud primitive fitting has always been a focal point of interest for researchers. This paper provides a comprehensive review of cutting-edge methods for 3D point cloud primitive fitting, with detailed technical trends referenced in in-depth research literature [8,9].

In traditional methods, the processing primarily involves RANSAC [10,11,12], parameter space transformation [13], and the use of clustering and segmentation techniques [14,15,16]. Among them, RANSAC [10] and its variant methods [11,17,18,19,20,21] are particularly common in the field of computer vision for the detection and fitting of geometric primitives. Methods based on RANSAC are capable of approximating the estimation of model parameters iteratively, demonstrating exceptional performance. However, this approach requires tedious parameter tuning for each primitive, which is also its application limitation. For example, Schnabel et al. [11] presented a robust framework based on RANSAC in their seminal paper, which effectively detects various geometric primitives in dense point clouds. Building on this, Li [17] introduced a post-optimization step, refining the relationship between the extracted primitives. Meanwhile, Wu et al. [9] and Du et al. [22] explored the application of RANSAC methods for reverse engineering of CSG models using input point clouds or mesh structures. Romanengo et al. [23] proposed a solution based on the Hough Transform that can identify simple geometric primitives and exhibits a certain degree of robustness to noise. Although these methods yield significant results in their application domains, their performance is often constrained by intricate parameter tuning. Moreover, the normal information generated during the point cloud processing is crucial for primitive fitting, yet it is not easily attainable in 3D scanning. In contrast, our deep-learning network requires no additional information during the testing phase and solely relies on the coordinates of the point cloud data to function.

In terms of approaches based on neural networks, Tulsiani et al. [24] and Zou et al. [25] were among the first to introduce neural networks for deconstructing and decomposing shapes. They proposed a network architecture that assembles complex geometric shapes using simple 3D voxels. Subsequent researchers have extended this idea. For instance, Sun et al. [26] constructed a network architecture for predicting cuboid parameters to abstract the structure of 3D objects. Smirnov et al. [27] introduced a framework for predicting parametrized shape primitives through deep learning, which utilizes distance fields to perform transformations between pixel grids and input data, applicable to 2D and 3D tasks. Lin et al. [28] proposed a method for editing primitive adaptations to target shapes based on reinforcement learning. Subsequent research focused on fitting various types of primitives, such as solutions based on super quadric geometric primitives studied by Paschalidou et al. [29]. Gadelha et al. [30] developed a generative model that establishes compact 3D expressions by predicting parameters for cubical and spherical meshes. Additionally, networks for learning 3D representations of convex shape sets through recursive spatial subdivision have been proposed by Chen et al. [31] and Deng et al. [32]. Local shape elements expressed using implicit functions have been explored by Genova et al. [33,34]. However, the types of primitives used in these studies are fairly limited, with most work focusing on abstracting input shapes using coarse prototypes. Some research has considered fitting multiple primitive types, such as a network proposed by Gopal et al. [35] that predicts Constructive Solid Geometry (CSG) structures from complex primitive geometric shapes, though its accuracy is limited by the definition within low-resolution voxel grids. Li et al. [36] proposed a geometric primitive-fitting network based on surface and edge detection, with an emphasis on learning edge and geometric features. However, the network may fail when edges of adjacent primitives are missing. Li et al. [5] presented the SPFN framework for more precise fitting of various primitives including planes, spheres, cylinders, and cones; Sharma et al. [37] further extended this method to adapt to B-spline surface stitching; Saporta [38] undertook unsupervised recursive structural modification of this framework; while Lê et al. [7] applied a cascaded structure combining local and global features to SPFN. Although these methods are effective, most of them use the ready-made network architecture PointNet++ [6] to encode input point clouds and are thus subject to constraints of the point cloud scale (such as processing at most an 8 k magnitude of points) and hindered by the limitations of point cloud feature extraction capabilities. Our proposed method innovatively provides a new network architecture composed of a preprocessing module, feature extraction module, classification segmentation module, and parameter prediction module.

## 3. Method

Our network architecture predominantly consists of four parts: the preprocessing module (Section 3.1), feature extraction network (Section 3.2), classification and segmentation module (Section 3.3), and parameter prediction module (Section 3.4).

### 3.1. Preprocessing Module

**Figure 2:**
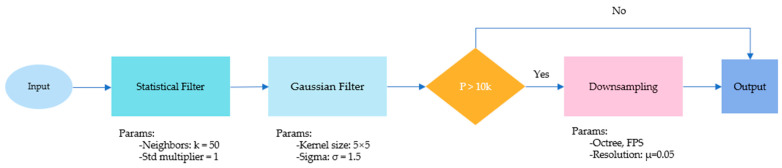
Flowchart of the preprocessing module. This module is utilized to filter out certain noise and to sample high-resolution point clouds.

The preprocessing module plays an indispensable role in the construction of deep-learning architectures. As shown in Figure 2, the core function of this module lies in optimizing the computational efficiency and overall performance of the network.

In the practical application of deep learning, particularly when dealing with complex 3D point cloud data, one common challenge we encounter is data noise. We first perform statistical filtering on the point set $\left\{x_{1},x_{2},\ldots ,x_{n}\right\}$, calculating the average distance $\left\{d_{1},d_{2},\ldots ,d_{n}\right\}$ of each point to its K nearest neighbors. Points satisfying $d_{i}>\mu +c\cdot \sigma$ are removed, resulting in the subset $\left\{x_{1},x_{2},\ldots ,x_{m}\right\}$, where μ and σ are the mean and standard deviation of the average distances of the point set, respectively, and c is the standard deviation multiplier. Then, for each point $P_{i}\in \left\{x_{1},x_{2},\ldots ,x_{m}\right\}$, Gaussian filtering is applied to reduce positional perturbations caused by noise with the following equation:

(1) $$P_{i}^{\prime }=\frac{\sum_{j=1}^{n}w_{ij}\cdot P_{j}}{\sum_{j=1}^{n}w_{ij}}$$

where $w_{ij}=e^{-\frac{d_{ij}^{2}}{2\sigma^{2}}}$ represents the Gaussian weight of point $P_{j}$ relative to point $P_{i}$, and $d_{ij}$ represents the distance between points $P_{i}$ and $P_{j}$.

**Figure 3:**
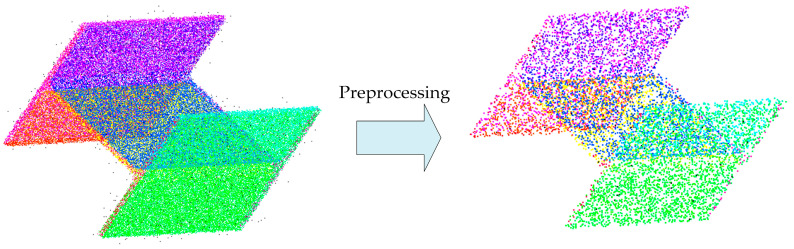
Enhancing point cloud data through noise reduction, smoothing, and down-sampling.

High-resolution three-dimensional point cloud data typically exhibit an extremely high data density, containing hundreds of thousands or even millions of data points. The high density of data points exerts significant pressure on computational resources and increases the complexity of processing. Therefore, it is essential to significantly alleviate computational and storage burden by reducing the total number of data points during the preprocessing stage while maintaining the core structural characteristics of the data. Consequently, our network automatically performs down-sampling on point clouds with a number of points exceeding 10k within the preprocessing module. Figure 3 is the visualization of the point cloud after being processed by the preprocessing module.

### 3.2. Feature Extraction Network (FEN)

**Figure 4:**
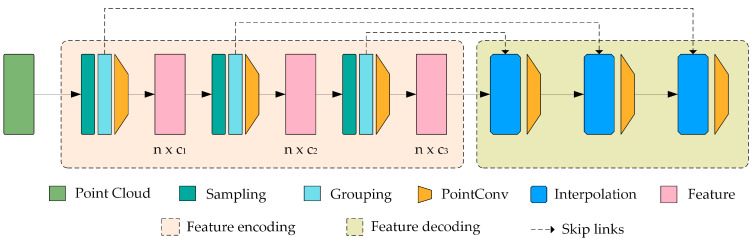
Feature extraction network architecture diagram. This network implements a novel feature aggregation layer based on hierarchical PointConv kernels.

Before delving into the role of the feature extraction network, it is necessary to clarify its core objectives and working principles. The primary task of the feature extraction network is to handle point cloud data containing multi-dimensional features, distilling key information that aids subsequent tasks. In this section, we will introduce the main components of the feature extraction network. Figure 4 depicts the network structure diagram, which adopts a hierarchical structure similar to that of PointNet++ [6].

#### 3.2.1. Input and Output

The feature extraction network accepts a matrix of size N×(d+C) as input, which represents a point cloud composed of N points, with each point containing not only d-dimensional spatial coordinates but also C-dimensional attribute features. After a series of operations performed by the network, it outputs another matrix of dimensions N′×(d+C′). In the output matrix, the number of points is reduced to N′, and each point retains its spatial coordinates while also acquiring an updated feature vector of dimension C′. In this manner, the network summarizes the local contextual information of each point, culminating in a more advanced representation of features.

#### 3.2.2. Sampling Layer

The input point cloud data are initially processed through a sampling layer, which extracts a representative subset $\left\{x_{1},x_{2},\ldots ,x_{m}\right\}\;$ from the initial set of points $\left\{x_{1},x_{2},\ldots ,x_{n}\right\}$. The traditional farthest point sampling (FPS) [39] algorithm experiences a linear increase in time complexity with the increase in the number of points, leading to significant time consumption when dealing with large-scale point cloud data. To address this issue, we applied the optimized farthest point sampling (OFPS) [40,41] algorithm, which accelerates the retrieval speed for the farthest points by incorporating a spatial data structure known as the KD-tree. It also significantly reduces the frequency of distance computations through a local search strategy. The core objective is to more effectively select key points from the point cloud that represent the overall distribution of the data, ensuring that the sampled points are evenly distributed in space to achieve high-quality coverage of the entire set of points.

#### 3.2.3. Grouping Layer

Following the sampling layer, the grouping layer utilizes the set of key points selected by the sampling layer as a set of centroids. Using a spherical neighborhood algorithm centered on these centroids, it interweaves the points from the original set that are relevant to them, thereby forming multiple local point sets. This layer operates on the input point set with dimensions N×(d+C), in combination with a set of centroid coordinates with dimensions N′×d, ultimately producing a group of point sets with dimensions N′×K×(d+C′). Each set of data represents a local neighborhood of a centroid, where K is the number of points within the neighborhood. Although the K values can vary across different groups, subsequent network layers can unify these local neighborhood features, with a varying number of points, into fixed-length feature vectors. In convolutional neural networks, the local region of pixels includes those whose array indices are within a certain Manhattan distance. In point sets sampled from metric spaces, the neighborhood of points is defined by the metric distance. Considering that the density of real-world-scanned point cloud data is often uneven, an adaptive neighborhood algorithm is employed. Specifically, the local point cloud density is estimated based on the distances to the K nearest neighbors of each key point, where the distance can be represented as the distance from the point to its *K*-th nearest neighbor, and then this distance is used as the adaptive radius for that point. In areas of high density, the search radius is smaller, as a few neighbors are sufficient to represent the local features of the region; in sparser areas of the point cloud, the search radius is increased to ensure that a sufficient number of neighboring points are captured, allowing the point cloud to automatically adapt to varying densities.

#### 3.2.4. PointConv Layer

Then, the network employs a PointConv [42] layer to emulate the effect of traditional two-dimensional convolution within a point cloud. Unlike two-dimensional convolution, which operates with a fixed kernel sliding over a pixel grid, PointConv functions within the local neighborhood of each point using a set of specific importance weight functions. These weight functions take into account the spatial position of points, existing features, and the local point density to calculate the contribution of each point in the feature-learning process. The following equation is used when calculating each point’s contribution to the feature learning process:

(2) $$F_{i}^{\prime }=\sum_{x_{j}\in H\left(x_{i}\right)}W\left(x_{i},x_{j}\right)F_{j}\frac{1}{\rho \left(x_{j}\right)}\Delta V_{j}$$

where $F_{j}$ represents the input features of point $x_{j}$, $F_{i}^{\prime }$ represents the output features, W is the weight function of the continuous convolution, $\rho (x_{j})$ is the density estimation of point $x_{j}$, $\Delta V_{j}$ represents the volume element, and $H(x_{i})$ denotes the neighborhood set of $x_{i}$. Through this approach, the integration of PointConv kernels with sampling and grouping can accommodate the non-uniform distribution of point cloud data and learn more enriched feature representations.

### 3.3. Segmentation and Classification Module (SCM)

**Figure 5:**
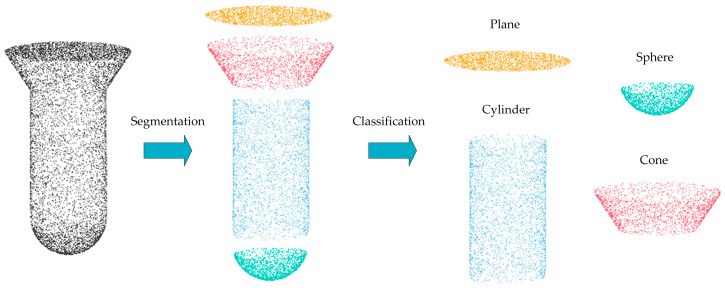
Segmentation and classification schematic. The point cloud is segmented by the network into individual constituent fragments, which are then classified according to geometric primitives.

As shown in Figure 5, the network performs segmentation on the point cloud after feature extraction, dividing it into several subsets. It performs classification on each segmented subset in order to determine its most compatible primitive geometric type (Figure 5: plane, sphere, cylinder, or cone). Inspired by the SPFN [5] approach, we compute the Relaxed Intersection over Union (RIoU) for all column pairs in the membership matrix *W* and $\tilde{W}$. The definition of RIoU is as follows:

(3) $$RIoU\left(W,\tilde{W}\right)=\frac{W⨀\tilde{W}}{W+\tilde{W}-W⨀\tilde{W}}$$

In this context, $W\in {\left\{0,1\right\}}^{N\times K}$ is a predefined ground truth matrix, and $\tilde{W}\in {\left[0,1\right]}^{N\times K}$ is the prediction matrix; ⨀ denotes the Hadamard product of matrices; the plus and minus signs represent the element-wise addition and subtraction of matrices, respectively. N is the total number of points, and K∈{0,1,2,3} represents different geometric primitives: planes, spheres, cylinders, and cones, respectively.

In the segmentation task, the network assigns a category label to each individual point in the point cloud, which requires the utilization of both local and global features. Local features are passed to the decoder and fused with global features to generate the predicted classification for each point. Here, the decoder comprises two components: interpolation and PointConv [42]. Initially, we employ an interpolation method to propagate the coarse features from the previous layer, interpolating from the features of the three nearest neighboring points through linear interpolation. Subsequently, with the use of skip-links, the interpolated features are combined with features generated by a convolutional layer of matching resolution. The interpolation satisfies the formula:

(4) $$v\left(q\right)=\frac{\sum_{i}^{k}w_{i}v_{i}}{\sum_{i}^{k}w_{i}+\varepsilon }$$

where $w_{i}=\frac{1}{d_{i}^{p}}$, with q being any query point, the initial value of p set to 2, and k set to 3; ε is a small constant to avoid the situation of division by zero, $v_{i}$ denotes the feature vector of the *i*-th point, and $d_{i}$ denotes the distance from the *i*-th point to the query point q. Meanwhile, skip connections update the feature vector of each point through a fully connected layer and a ReLU layer, ensuring the effective transmission of features and non-linear enhancement. After combining the features, we apply the PointConv operation on the mixed features to obtain the final deconvolution output, a process similar to deconvolving layers in image processing. This procedure is repeated until the features of all the input points are propagated back to the original resolution.

In classification tasks, the network predicts the category of the primitive type to which the input point cloud belongs. Local features are extracted from points within a neighborhood through multiple small PointConv networks. Then, a max-pooling operation is performed on all points to obtain a global feature $F_{G}$ representation. By feature aggregation, the network is able to synthesize attributes from different spatial positions and thus consolidate them into a global feature representation via global max-pooling. This global feature representation is subsequently fed into a multi-layer perceptron (MLP) to capture the complex relationships between global features and to make classification judgments based on the extracted features.

### 3.4. Parameter Prediction Model

**Figure 6:**
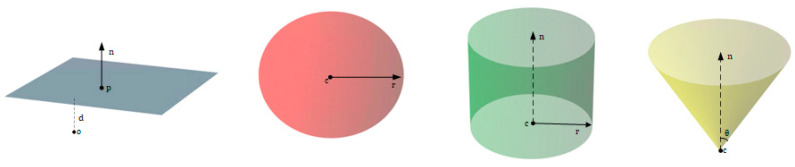
Basic primitives and parameters.

Building upon the identification of geometric primitives for each segment by the network, as described in the previous section (Refer to Figure 6, where labels (0, 1, 2, 3) are used to denote a plane, sphere, cylinder, and cone, respectively.), the function of this network component is to predict the precise mathematical parameters corresponding to these primitives.

#### 3.4.1. Plane

The fitted plane can be represented by the following equation:

(5) $$N^{T}x+d=0$$

where N is the normal vector of the plane, and d is the distance to the origin. Our goal is to find the vector N and the scalar d that minimize the weighted least squares error function:

(6) $$L\left(N,d\right)=\sum_{i=1}^{N}w_{i}(N^{T}P_{i}+d{)}^{2}$$

Here, the error is the distance from a point P to the plane, and $w_{i}$ represents the weight, which indicates greater consideration given to the point when the weight is larger. To solve this problem, we take the partial derivatives with respect to N and d, set them to zero, and obtain the linear system of equations:

(7) $$G^{T}WGH=b_{1}$$

where $G=[P_{i}^{T},1]$, $H={\left[N,d\right]}^{T}$, and $b_{1}$ is a zero vector. The system of equations is solved by finding the solution H through singular value decomposition (SVD).

#### 3.4.2. Sphere

The fitted sphere can be described by the following equation:

(8) $$F_{sphere}=\left(c_{1},r\right)$$

where $c_{1}=(c_{x},c_{y},c_{z})$ is the center of the sphere, and r is the radius of the sphere. Given a set of points P and corresponding weights w, the optimal sphere is found by minimizing the sum of squared weighted distances, given by the following equation:

(9) $$L^{2}\left(w,c_{1},r\right)=\sum_{i=1}^{N}w_{i}{\left({\left|\left|P_{i}-c_{1}\right|\right|}^{2}-r^{2}\right)}^{2}$$

Setting $\frac{\partial F}{\partial r^{2}}=0$ yields $r^{2}=\frac{{\sum_{1}^{N}w_{i}\left\|P_{i}-c_{1}\right\|}^{2}}{\sum_{i=1}^{N}w_{i}}$. Substituting $r^{2}$ into the equation above yields the following equation in terms of $c_{1}$:

(10) $$F\left(w,c_{1}\right)={\left\|{\left(diag\left(w\right)\right)}^{\frac{1}{2}}\left(Xc_{1}-y\right)\right\|}^{2}$$

Let the centroid of all points be $\bar{P}=\frac{\sum_{i=1}^{N}w_{i}P_{i}}{\sum_{i=1}^{N}w_{i}}$, where the *i*-th row of matrix X is $X_{i}=2(-P_{i}+\bar{P})$, and the i-th element of vector y is $y_{i}=P_{i}^{T}P_{i}-\frac{\sum_{i=1}^{N}w_{i}P_{i}^{T}P_{i}}{\sum_{i=1}^{N}w_{j}}$. Construct the coefficient matrix of the normal equation: $B=X^{T}X$, and the constant term: $b_{2}=X^{T}y$. Use Cholesky decomposition to solve the normal equation $Bc_{1}=b_{2}$ to find the sphere’s center $c_{1}$. Once we have the center $c_{1}$, we can calculate the square of the radius: $r^{2}=\frac{{\sum_{i=1}^{N}w_{i}\left\|P_{i}-c_{1}\right\|}^{2}}{\sum_{i=1}^{N}w_{i}}$.

#### 3.4.3. Cylinder

The parametric equation representing the fitted cylinder can be expressed as follows:

(11) $$F_{cylinder}=\left(\eta ,c_{2},r\right)$$

where the vector $\eta \epsilon R^{3}$ denotes the unit vector in the direction of the cylinder’s axis, the coordinate $c_{2}\epsilon R^{3}$ represents the center point of the cylinder, and $r\epsilon R^{3}$ is the radius of the cylinder. For a point P in space, we can compute the square of its distance to the cylinder’s surface, which is given by the following expression:

(12) $$D_{cylinder}^{2}={\left(\sqrt{{v_{1}}^{T}v_{1}-{\left(\eta^{T}v_{1}\right)}^{2}}-r\right)}^{2}$$

where $v_{1}=P-c_{2}$ represents the position vector of point P relative to the cylinder’s center $c_{2}$.

However, it is not straightforward to directly calculate this distance, as it involves non-linear operations. Therefore, to simplify the problem, we usually address the cylindrical fitting issue in two steps: First, we determine the cylinder’s axis vector. This can be achieved by finding a common direction in the set of the normals to the data points. The normal N on the data point is perpendicular to the axis vector of the cylinder. Therefore, we look for a vector η such that the dot product of the normals of all points with this vector is minimized as much as possible.

(13) $$\xi_{cylinder}={\left\|\sqrt{diag\left(w\right)}N\eta \right\|}^{2}$$

Then, once the axis vector η has been identified, we can determine the cylinder’s center point $c_{2}$ and radius r. We assume that all data points form a circular shape when projected onto the plane perpendicular to the axis of the cylinder. We fit a circle through these projected points, a step very similar to fitting a sphere, where the objective is to find the optimal center and radius that best approximate the data points.

#### 3.4.4. Cone

The cone can be parameterized as follows:

(14) $$F_{cone}=\left(\eta ,c_{3},\theta \right)$$

where η is a unit vector, denoting the axis direction emanating from the vertex $c_{3}$ of the cone, and $\theta \in (0,\frac{\pi }{2})$ is the semi-angle of the cone. The square of the distance to the cone’s surface is given by the following equation:

(15) $$D_{cone}^{2}={\left(\left\|v_{2}\right\|\sin\left(\min\left(\left|\eta -\theta \right|,\frac{\pi }{2}\right)\right)\right)}^{2}$$

where $v_{2}=P-c_{3}$, $\eta =arccos(\frac{\eta^{T}v_{2}}{\left\|v_{2}\right\|})$. When solving the computational problem of approximating the cone’s vertex and axis, we first independently estimate η and $c_{3}$ and then estimate the semi-angle θ. Since all the intersection points of the tangential planes on the cone’s surface are the vertex $c_{3}$ of the cone, the multiplane intersection problem can be represented as a least squares method by using the predicted normal vectors N.

(16) $$\xi_{cone}\left(c_{3},N\right)={\left\|{\left(diag(w\right))}^{\frac{1}{2}}\left(Nc_{3}-y\right)\right\|}^{2}$$

where $y_{i}=N_{i}^{T}P_{i}$. Finally, with the vertex c and the axis vector η, the semi-angle θ can simply be calculated as a weighted average: $\theta =\frac{1}{\sum_{i=1}^{N}w_{i}}\sum_{i=1}^{N}w_{i}arccos\left|\eta^{T}\frac{P_{i}-c_{3}}{\left\|P_{i}-c_{3}\right\|}\right|$.

### 3.5. Evaluation Metrics

We employ the following metrics [4] to evaluate our network:

1. Segmentation mean intersection over union (IoU)

This metric is used to measure the similarity between the predicted segmentation and the true segmentation. It is defined as follows:

(17) $$S=\frac{1}{k}\sum_{i=1}^{K}IoU\left({\tilde{W}}_{:,i},h\left(W_{:,i}\right)\right)$$

where h denotes the conversion to a one-hot vector representation, *K* is the number of true segments, and for details on IoU, please refer to Section 3.3.

2. Mean primitive type accuracy

This metric is used to measure the average accuracy of predicted primitives. It is defined as follows:

(18) $$T=\frac{1}{K}\sum_{i=1}^{K}1\left[t_{i}={\tilde{t}}_{i}\right]$$

where 1 is the indicator function, and $t_{i}$ and ${\tilde{t}}_{i}$ are the true and predicted primitive types for the *i*-th segment of the point cloud, respectively.

3. Mean point normal difference

This metric is used to measure the discrepancy between predicted normal vectors and true normal vectors, defined as follows:

(19) $$N=\frac{1}{K}\sum_{i=1}^{N}\arccos\left(\left|N_{i}^{T}{\tilde{N}}_{i}\right|\right)$$

where $N_{i}$ and ${\tilde{N}}_{i}$ are the true and predicted normal vectors for the *i*-th point, respectively.

4. Mean primitive axis difference

This metric is utilized to compare the difference between the predicted primitive axes and the true axes, with lower values indicating higher precision in orientation prediction. It is calculated as follows:

(20) $$A=\frac{1}{\sum_{i=1}^{K}1\left[t_{i}={\tilde{t}}_{i}\right]}\sum_{i=1}^{K}[t_{i}={\tilde{t}}_{i}]\arccos\left(\cos\left(\Theta \left(\eta_{i},{\tilde{\eta }}_{i}\right)\right)\right)$$

where $\eta_{i}$ and ${\tilde{\eta }}_{i}$ are the true and predicted principal axes of the *i*-th primitive, respectively, and cos(Θ) represents the cosine of the angle between two axes.

5. Mean residual

The average distance residual of actual data points to the surface of the predicted primitive. It is computed as follows:

(21) $$R=\frac{1}{K}\sum_{i=1}^{K}E_{p\sim U\left(S_{i}\right)}D\left(p,{\tilde{A}}_{i}\right)$$

where $E_{p\sim U\left(S_{i}\right)}$ is the expectation over points p sampled uniformly from the surface $S_{i}$ of the true primitive, and $D(p,{\tilde{A}}_{i})$ is the distance from p to the predicted primitive surface ${\tilde{A}}_{i}$.

6 P coverage

This metric represents the proportion of points in the point cloud that are covered by the predicted geometric primitives. It is defined as follows:

(22) $$C=\frac{1}{N}\sum_{i=1}^{N}I\left({min}_{j=1}^{K}\left(\sqrt{D\left(P_{i},{\tilde{A}}_{j}\right)}\right)<\varepsilon \right)$$

where $P_{i}$ is the *i*-th point in the point cloud, ${\tilde{A}}_{j}$ denotes the surface of the *j*-th predicted primitive, $D\left(P_{i},{\tilde{A}}_{j}\right)$ is the square of the Euclidean distance from $P_{i}$ to ${\tilde{A}}_{j}$, ε is a predefined threshold, and I is the indicator function, which values at 1 if the condition inside the parenthesis is true, and 0 otherwise.

## 4. Experiments

**Figure 7:**
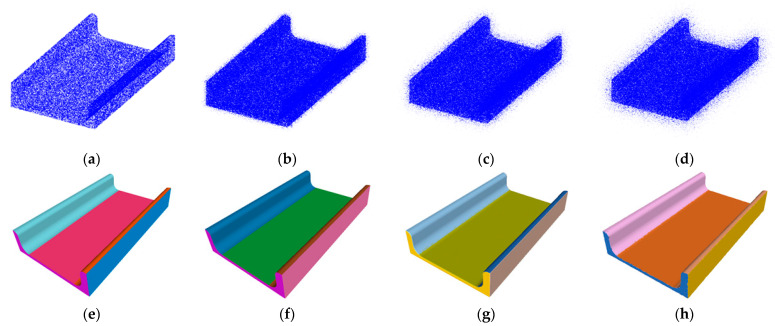
Noise simulation by adding Gaussian noise with standard deviations: (**a**) original data, (**b**) 5% noise, (**c**) 10% noise, and (**d**) 15% noise, (**e**–**h**) corresponding to the respective fitted results.

We utilized CAD models of mechanical parts that conform to the American national standards institute (ANSI) standards, provided by Traceparts [5], to train and assess the network we proposed. The training dataset contains 13,831 models, while the validation and test datasets each contain 3366 models, with each model consisting of 131,072 points. The training process was conducted on a computer equipped with an Intel Xeon(R) Silver 4210R CPU and an NVIDIA GeForce RTX 3090 GPU. During the training process, the initial learning rate is set to 0.001, with a decay rate of 0.7, and the learning rate is decayed every 200,000 steps. The batch size is 32, and the total number of training epochs is 300. The weights for the loss functions, including MIoU loss, normal loss, type loss, parameter loss, residue loss, and total loss, are all set to 1.0 to ensure that all loss components have an equal impact on the model. Meanwhile, the weight for the orthogonality loss is set to 0.1 to enhance the geometric properties of the model. Validation is performed every 2000 steps, and model snapshots are saved every 500 steps. The supported primitives include spheres, planes, cylinders, and cones. To ensure the rigor of our experiments, all models in the dataset underwent normalization such that their centroids were positioned at the origin of the coordinate system. In an effort to examine and enhance the performance of the network in noisy environments and improve the robustness of the algorithm, we simulated noise that might be encountered when receiving data from real sensors by adding Gaussian noise with standard deviations of 5%, 10%, and 15% to the original data, which is displayed in Figure 7 as an example. With this approach, our aim is to bring the development and testing environments as close as possible to real-world application scenarios, thereby increasing the practical value of our research.

We compared the performance of the method proposed in this study with the efficient RANSAC algorithm [11] in terms of primitive fitting, where RANSAC used the default adaptive algorithm parameters. Table 1 details the experimental results of our network versus efficient RANSAC.

**Figure 8:**
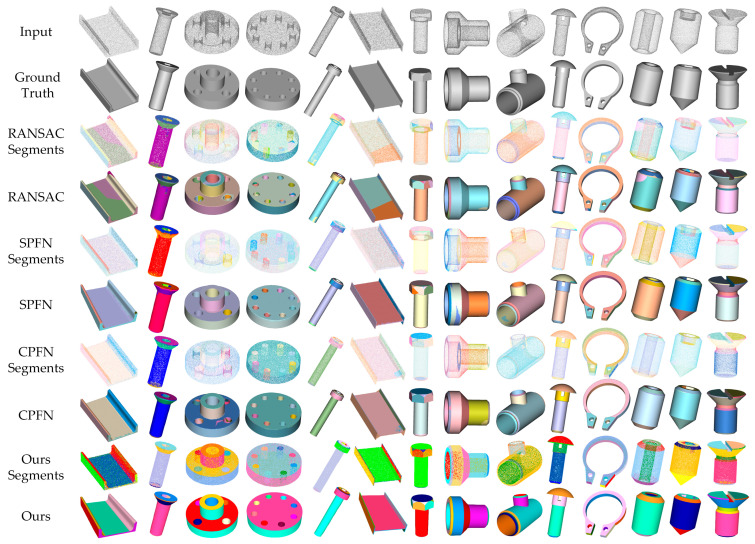
Primitive segmentation and surface reconstruction results.

Observations from the data reveal that compared to the RANSAC algorithm, our network achieved a significant improvement in mean intersection over union, increasing from 55.81% to 87.30%. This leap markedly demonstrates the advantage of our network in point cloud segmentation precision. In terms of geometric primitive type recognition, the accuracy of RANSAC was 63.03%, while our network significantly increased it to 95.62%, substantially enhancing the ability to recognize complex geometric shapes. For the estimation error of point normals and primitive axes, our method achieved 7.68 and 1.62, respectively, which were clearly superior to RANSAC’s 11.36 and 4.98, reflecting our algorithm’s greater precision in geometric feature estimation. Furthermore, the residual decreased to 0.002 and coverage increased to 91.76%, indicating that our method has a more advantageous understanding and representation of 3D point cloud data in terms of accuracy and comprehensiveness. Furthermore, Figure 8 demonstrates that our method generates segmentation results and models of exceptional quality that closely mirror the ground truth, outperforming all preceding techniques.

In comparison with the SPFN method, our network increased the Mean IoU from 75.66% to 87.30%, further validating our method’s superiority in maintaining high segmentation quality. In terms of accuracy for primitive-type recognition, our method also improved from SPFN’s 88.73% to 95.62%, significantly strengthening the classification performance for different 3D geometric primitives. Although SPFN had already achieved low values in point normal and primitive axis estimation errors (1.67 and 0.004, respectively), our network still accomplished further optimization in these metrics, reducing residuals to 1.62 and 0.002, showcasing an even more outstanding performance. The enhancement in point cloud coverage also highlights the comprehensiveness of our algorithm in integrated geometric information processing. When compared with the CPFN method, our network demonstrated a significant advantage in accurately segmenting point cloud data, with an increase in the mean intersection over union (IoU) from 78.15% to 87.30%. In terms of primitive-type recognition, the accuracy of our network improved from 93.56% to 95.62%, which underscores the network’s precision in identifying a diverse range of geometric primitives. Even though the numerical results are close to those of CPFN in point normal and primitive axis estimation, our network still exhibited further optimization by reducing the error to a very low level. By decreasing the residual to only 0.002 and improving the point cloud coverage to 91.76%, our network has effectively proven its exceptional capability in capturing the complexity of point cloud data in great detail.

**Figure 9:**
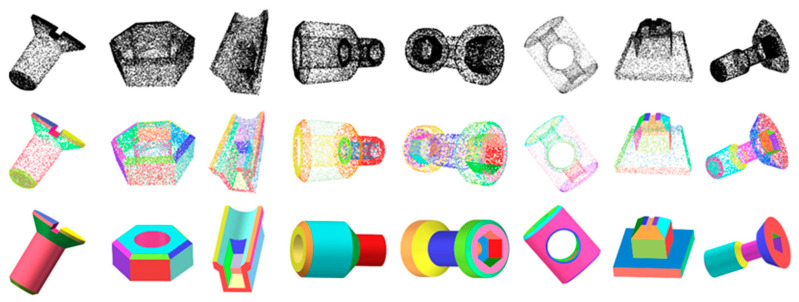
Primitive fitting of CAD models. Top-to-bottom rows: initial points, point-to-primitive assignment (denoted by distinct colors), and primitive fitting.

Figure 9 displays the fitting results of the neural network’s predictions for the geometric primitive parameters applied to CAD models. The input point cloud is displayed at the top of the image. The middle row shows the color-coded representation of the point cloud after our network predicts the point-to-primitive affiliations, where points of the same color represent classification to the same geometric primitive. The bottom line presents three-dimensional primitive graphic meshes assembled by a sequence of CSG [35] operations, which are based on parameters predicted by our network. 

These CSG operations, such as union, intersection, and subtraction, are implemented with user assistance. In this study, we treat the CSG sequence as cases of multiple primitives overlapping, and it becomes necessary to determine the appropriate sequence of CSG operations. As the number of overlapping primitives increases, the number of potential CSG sequences escalates exponentially. To circumvent the exponential complexity of searching for the correct sequence of CSG operations, we allow users to manually determine the suitable operation between primitives, be they union, intersection, or subtraction. Therefore, in situations where multiple primitives intersect, users are required to manually select the primitives and assign the appropriate CSG operations, such as union, intersection, or subtraction.

Additionally, we conducted an experiment to compare the execution times of the four aforementioned methods using point cloud data with a size of 131,072. The traditional RANSAC algorithm, using default parameters, had an execution time of 0.542 s on a CPU. For machine-learning-based methods, we evaluated pre-trained models without including training time (which takes several days). The execution times on a GPU were as follows: 0.035 s for SPFN, 0.039 s for CPFN, and 0.058 s for our method. These results indicate that our proposed method also demonstrates competitive execution times, enabling the rapid generation of experimental results.

To corroborate the precision of our network in predicting geometric primitive parameters, we utilized the point cloud library (PCL) [43] to efficiently generate three-dimensional point cloud data for four typical regular surfaces commonly found in industrial products, such as planes, spheres, cylinders, and cones. The dataset for each type of surface contained 60,000 points, and we injected different levels of Gaussian noise into all of the three-dimensional point cloud data to mimic the characteristics of point cloud data obtained from laser scanning in the real world.

In the case of planar entities, we generated 1000 3D plane point cloud datasets that satisfy the normal vector n=(1,0,0) and a distance d=1 from the origin. Table 2 shows the predicted results of the primitive parameters after adding different noise levels (no noise, 5% noise, 10% noise, 15% noise) and the absolute error compared to the theoretical ground truth.

(23) $$Absoulte\;Error=\sum_{i=1}^{N}\left|\lambda_{i}-\lambda_{i}^{\prime }\right|$$

where λ represents the ground true parameter, $\lambda^{\prime }$ represents the predicted parameter, and N is the total number of parameters. From the table above, it can be observed that even after the introduction of noise, our network still retains the ability to predict the geometric parameters of the surfaces with a high degree of accuracy, demonstrating its robustness in handling noisy point cloud data.

For spherical entities, we created 1000 point cloud datasets of spheres centered at $\left(0,0,0\right),$ with a radius of 6, incorporating varying degrees of Gaussian noise. Table 3 presents the predicted spherical parameters at different noise levels (no noise, 5%, 10%, and 15%) and their absolute errors relative to the theoretical ground truth. The data indicate that, despite noise interference, our network retains proficiency in parameter estimation for spheres. Remarkably, with 15% noise, absolute errors remain well contained, validating our network’s precision and resilience in handling noisy point cloud data.

For cylindrical entities, we generated 1000 cylindrical point cloud datasets with an axial vector n=(0,0,1), a center point c=(0,0,0), and a radius of 6. Table 4 presents the predicted cylindrical parameters with various levels of noise (no noise, 5%, 10%, 15%) and the corresponding absolute errors relative to the theoretical ground truth. The results, as evidenced by the table, show that our network proficiently predicts the parameters of cylinders, despite the presence of noise interference.

In the case of conical entities, we generated 1000 conical point cloud datasets with the apex c=(0,0,0), axial vector n=(0,0,1), and cone half-angle $\theta =\frac{\pi }{4}$. Table 5 shows the predicted results of the conical parameters after adding different levels of noise (no noise, 5% noise, 10% noise, 15% noise), along with the absolute error, compared to the theoretical ground truth. It can be observed that despite the presence of noise interference, our network still demonstrates good performance in predicting the parameters of conical surfaces.

## 5. Conclusions

In the field of computer vision, point cloud segmentation and geometric primitive recognition are fundamental and crucial upstream tasks. This study is dedicated to proposing an innovative neural network architecture that exhibits outstanding performance in the precise segmentation and robust recognition of geometric primitives for 3D point cloud data. A series of stringent experiments conducted have verified the excellence in accuracy of our proposed neural network over existing technologies when addressing issues of point cloud segmentation and geometric primitive recognition. Specifically, our network has achieved significant improvements in the accuracy of predicting parameters of geometric primitives, which will offer a new alternative approach in applications related to 3D geometric modeling and object recognition. In addition, given the high potential and versatility displayed by this network in complex computer vision tasks, it not only enhances the insight of computer vision systems into three-dimensional space but also offers powerful tools for various fields to more effectively parse and utilize 3D spatial data. Focusing on improving the precision of point cloud segmentation not only directly affects the accuracy of geometric primitive parameter estimation but is also a prerequisite for understanding complex 3D structures. Looking ahead, we plan to further evaluate our methods using real-world data collected by actual 3D sensors. In addition, beyond planes, cylinders, spheres, and cones, we will also explore extending our current approaches to more generic shapes and primitives.

## Figures and Tables

**Table 1:** Results of all experiments. The bold values in the table represent the best results.

Method	Seg. (Mean IoU) ↑	Type Accuracy ↑	Point Normal ↓	Primitive Axis ↓	Residual ↓	P Coverage ↑
RANSAC	55.81	63.03	11.36	4.98	0.058	81.21
SPFN	75.66	88.73	9.43	1.67	0.004	88.13
CPFN	78.15	93.56	8.83	**1.56**	0.034	89.06
Ours	**87.30**	**95.62**	**7.68**	1.62	**0.002**	**91.76**

**Table 2:** Parameter of plane primitive. Prediction parameters and absolute errors of point clouds of planar primitives under different noises.

Plane	Ground-Truth	Noise-Free	5% Noisy	10% Noisy	15% Noisy
Normal_x	1.000000	1.000000	0.999989	0.999998	0.999983
Normal_y	0	0	−0.002726	−0.002029	−0.001594
Normal_z	0	0	0.001895	0.000851	0.005571
d	1	1	0.998976	1.014328	1.085687
Absolute Error	-	0	0.005656	0.017210	0.092869

**Table 3:** Parameter of sphere primitive. Prediction parameters and absolute errors of point clouds of sphere primitives under different noises.

Sphere	Ground-Truth	Noise-Free	5% Noisy	10% Noisy	15% Noisy
Center_x	0.000000	0.000000	0.004954	0.029597	0.011205
Center_y	0.000000	0.000000	0.000071	0.009223	0.015344
Center_z	0.000000	0.000000	0.003963	−0.017266	−0.012764
r	6.000000	6.000000	6.002710	6.096920	6.217777
Absolute Error	-	0	0.011698	0.153006	0.257090

**Table 4:** Parameter of cylinder primitive. Prediction parameters and absolute errors of point clouds of cylinder primitives under different noises.

Cylinder	Ground-Truth	Noise-Free	5% Noisy	10% Noisy	15% Noisy
Axis_x	0.000000	0.000000	0.001241	0.004835	−0.001827
Axis_y	0.000000	0.000000	0.000197	−0.002319	0.001084
Axis_z	1.000000	1.000000	0.993506	0.897432	0.932910
Center_x	0.000000	0.000000	0.000327	0.005059	0.008748
Center_y	0.000000	0.000000	−0.000215	−0.006206	−0.000155
Center_z	0.000000	0.000000	−0.002756	0.153001	−0.344465
r	6.000000	6.000000	6.005907	6.010655	6.076211
Absolute Error	-	0	0.017137	0.284643	0.499580

**Table 5:** Parameter of cone primitive. Prediction parameters and absolute errors of point clouds of cone primitives under different noises.

Cone	Ground-Truth	Noise-Free	5% Noisy	10% Noisy	15% Noisy
Axis_x	0.000000	0.000000	0.001528	0.001570	0.018011
Axis_y	0.000000	0.000000	0.000161	0.002301	0.000327
Axis_z	1.000000	1.000000	0.999618	1.000382	0.989207
C_x	0.000000	0.000000	0.000270	0.001648	0.011430
C_y	0.000000	0.000000	0.000261	0.000741	0.004834
C_z	0.000000	0.000000	0.000503	0.000653	0.001576
θ	0.785398	0.785398	0.753899	0.873067	0.633701
Absolute Error	-	0	0.034604	0.094964	0.198668

## Data Availability

Data will be made available on request.
